# Evaluation on the Intrinsic Physicoelectrochemical Attributes and Engineering of Micro-, Nano-, and 2D-Structured Allotropic Carbon-Based Papers for Flexible Electronics

**DOI:** 10.1021/acs.langmuir.1c02121

**Published:** 2021-12-03

**Authors:** Supatinee Kongkaew, Lingyin Meng, Warakorn Limbut, Proespichaya Kanatharana, Panote Thavarungkul, Wing Cheung Mak

**Affiliations:** † Biosensors and Bioelectronics Centre, Division of Sensor and Actuator Systems, Department of Physics, Chemistry and Biology, Linköping University, SE-581 83 Linköping, Sweden; ‡ Center of Excellence for Trace Analysis and Biosensor, Prince of Songkla University, Hat Yai, Songkhla 90110, Thailand; § Center of Excellence for Innovation in Chemistry, Faculty of Science, Prince of Songkla University, Hat Yai, Songkhla 90110, Thailand; ∥ Division of Physical Science, Faculty of Science, Prince of Songkla University, Hat Yai, Songkhla 90110, Thailand; ⊥ Division of Health and Applied Sciences, Faculty of Science, Prince of Songkla University, Hat Yai, Songkhla 90110, Thailand

## Abstract

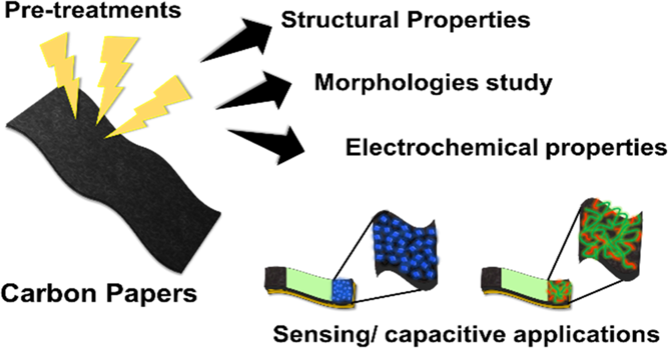

Flexible electronics have gained more attention for emerging electronic devices such as sensors, biosensors, and batteries with advantageous properties including being thin, lightweight, flexible, and low-cost. The development of various forms of allotropic carbon papers provided a new dry-manufacturing route for the fabrication of flexible and wearable electronics, while the electrochemical performance and the bending stability are largely influenced by the bulk morphology and the micro-/nanostructured domains of the carbon papers. Here, we evaluate systematically the intrinsic physicoelectrochemical properties of allotropic carbon-based conducting papers as flexible electrodes including carbon-nanotubes-paper (CNTs-paper), graphene-paper (GR-paper), and carbon-fiber-paper (CF-paper), followed by functionalization of the allotropic carbon papers for the fabrication of flexible electrodes. The morphology, chemical structure, and defects originating from the allotropic nanostructured carbon materials were characterized by scanning electron microscopy (SEM) and Raman spectroscopy, followed by evaluating the electrochemical performance of the corresponding flexible electrodes by cyclic voltammetry and electrochemical impedance spectroscopy. The electron-transfer rate constants of the CNTs-paper and GR-paper electrodes were ∼14 times higher compared with the CF-paper electrode. The CNTs-paper and GR-paper electrodes composed of nanostructured carbon showed significantly higher bending stabilities of 5.61 and 4.96 times compared with the CF-paper. The carbon-paper flexible electrodes were further functionalized with an inorganic catalyst, Prussian blue (PB), forming the PB-carbon-paper catalytic electrode and an organic conducting polymer, poly(3,4-ethylenedioxythiophene) (PEDOT), forming the PEDOT-carbon-paper capacitive electrode. The intrinsic attribute of different allotropic carbon electrodes affects the deposition of PB and PEDOT, leading to different electrocatalytic and capacitive performances. These findings are insightful for the future development and fabrication of advanced flexible electronics with allotropic carbon papers.

## Introduction

Flexible electronics have gained considerable attention for the development of newly emerging flexible and wearable electronic devices, including sensors and biosensors, batteries and capacitors, E-electronics and displays, etc., owing to their advantageous physicoelectrochemical properties including being lightweight, flexible, thin, and low-cost.^1^ Among all of the components in flexible electronics, the fabrication of flexible electrodes is crucially important. Current methods for fabrication of flexible electrodes can mainly be classified into the “bottom-up approach” such as wet chemistry based on the printing of conducting precursor inks or deposition of a conducting layer onto a flexible substrate^2,3^ and the “top-down approach” via shaping of a bulk prefabricated conducting flexible substrate into electrodes (e.g., carbon or metal sheet). For the wet-chemistry approach, various mask-based and mask-free printing techniques such as gravure printing, screen-printing, stencil printing, and inkjet printing were developed for the deposition of different conducting inks onto a substrate to create patterned electrodes.^4^ However, the wet-chemistry approach based on the printing of conducting inks has several limitations such as the extensive use of solvents for ink formulation, the requirement of an additional screen, stencil, or mask to pattern the conducting inks (except for inkjet printing), and a high-temperature ink curing process,^5^ while the vapor evaporation methods require expensive instruments for flexible electrode fabrication. Alternatively, other additive manufacturing approaches, including 3D printing,^6^ pencil drawing,^7^ and laser-direct writing,^8,9^ has been introduced for electrode fabrication. In contrast, the top-down approach using a prefabricated conducting flexible substrate that was produced in large scale by casting/assembling of nano-/microscopic conducting materials into macroscopic paperlike substrate offers a new route for the facile fabrication of advanced flexible electronic devices.^10,11^

Impacted by nanotechnology, the electrochemical properties of electronic or bioelectronic devices were largely improved by utilizing advanced allotropic materials such as various forms of metal nanoparticles (NPs) and nanorods;^12−15^ nanostructured and two-dimensional (2D)-structured carbon-based materials;^16−19^ and nanostructured conducting polymers.^20−23^ Among all, carbon-based materials (e.g., graphite, glassy carbon, carbon fiber) are the most commonly used electrode materials. Conventional carbon has many advantageous properties such as good electrical and thermal conductivities, good chemical stability, biocompatibility, wide operational window potential, and low background current.^24−26^ Beyond that, advanced nanostructured allotropic carbon such as carbon nanotubes and graphene with enhanced conductivity, faster electron-transfer rate, and higher surface area with improved electrode kinetics are promising materials for emerging electronic device applications.^27,28^ Furthermore, advanced organic and inorganic materials, such as ZnP nanosheets,^9^ metal nanoparticles,^29,30^ nickel sulfide nanocomposites,^31^ and poly(3,4-ethylenedioxythiophene) (PEDOT),^32^ were incorporated with allotropic carbon electrodes with improved conductivity and capacitive and electrochemical sensing performance.

Driven by the emerging conducting paper industry, great efforts have been devoted to the development of allotropic carbon papers with light weight and flexibility, such as carbon-fiber paper (CF-paper), carbon nanotubes paper (CNTs-paper, also known as bucky paper), and graphene-paper (GR-paper), as electrodes for various application fields including supercapacitors,^33^ lithium-ion batteries,^34^ electrocatalysis,^35^ and sensors and biosensors.^11^ The use of these prefabricated conducting papers does not require wet chemistry or an additional mask for printing and high-temperature curing processes, while the fabrication of the flexible electrode can be achieved by simple cutting/xurography and paper assembling. For the development of high-performance carbon-paper-based flexible electrodes, it is important to understand the intrinsic nano-/microscale characteristics of carbon nanotubes, graphene, and carbon fiber within the assembled bulk conducting papers such as morphology, chemical characteristic, and defects originating from allotropic nano-/microstructured carbon materials, as well as the mechanical bending stability, electrochemical properties, and their price-to-performance aspect so as to provide a comprehensive study on the utilization of conducting papers for the development of various flexible electronics.

Here, we aim to perform a comprehensive evaluation of the intrinsic physicoelectrochemical properties of allotropic carbon-based conducting papers as flexible electrode platforms. We further demonstrate the engineering and assembling of nano-/microstructured carbon papers for the development of flexible electrodes for sensing and energy applications. The morphology and the chemical structure of different allotropic carbon papers including CNTs-paper, GR-paper, and CF-paper were characterized, followed by systematic evaluation and mapping of their morphology and structural properties with the electrochemical performance characterized by cyclic voltammetry (CV) and electrochemical impedance spectroscopy (EIS). We also studied the mechanical bending stability of allotropic carbon papers as flexible electrodes. We demonstrated the postmodification performance of allotropic carbon papers with an inorganic catalyst, Prussian blue (PB), forming the PB-carbon-paper electrode for electrochemical sensing applications and an organic conducting polymer, poly(3,4-ethylenedioxythiophene) (PEDOT), forming the PEDOT-carbon-paper electrode for energy applications.

## Experimental Section

### Materials

Disodium hydrogen phosphate monohydrate (Na_2_HPO_4_·H_2_O) and potassium dihydrogen phosphate (KH_2_PO_4_) were obtained from Merck (Darmstadt, Germany). Hydrogen peroxide (H_2_O_2_), potassium ferrocyanide (K_4_[Fe(CN)_6_]), hydrochloric acid (HCl), isopropanol ((CH_3_)_2_CHOH), potassium ferricyanide (K_3_[Fe(CN)_6_]), potassium chloride (KCl), iron(III) chloride (FeCl_3_), 3,4-ethylenedioxythiophene (EDOT), and poly(styrene sulfonate) (PSS) were purchased from Sigma-Aldrich (Louis). All chemicals were of analytical reagent grade. The phosphate buffer solution (PBS, 0.1 M, pH 7.4) was prepared by mixing a stock solution of KH_2_PO_4_ and Na_2_HPO_4_. All solutions were prepared using deionized water (Milli-Q purification system, MerckMillpore, MA). CNTs-paper (Bucky paper, 60 GSM, measured thickness 356 ± 6 μm, weight per area 5.904 ± 0.005 mg cm^–2^) was purchased from Nanotech Lab (NC). GR-paper (measured thickness 195 ± 9 μm, weight per area 21.70 ± 0.01 mg cm^–2^) was purchased from Sigma-Aldrich (Louis). CF-paper (measured thickness 188 ± 2 μm, weight per area 8.56 ± 0.00 mg cm^–2^) was purchased from FullCellStore (TX). Plastic sheet and double-sided adhesive tape were purchased from Biltema (Linköping, Sweden).

### Activation of Allotropic Conducting Carbon Papers

Two different techniques were employed for the activation of allotropic conducting carbon papers including chemical treatment with isopropanol and physical treatment with oxygen plasma. In brief, for chemical pretreatment, carbon papers were immersed in isopropanol for 10 min, followed by drying inside an incubator at 100 °C for 1 h. In this process, the excessive surfactant or impurity contamination that comes from the manufacturing can be eliminated, exposing the original carbon-based material properties.^36^ For physical pretreatment, carbon papers were activated with oxygen plasma for 1 min (Diener electronic, Plasma Surface Technology, Pico, Germany). In this process, the free radicals, ions, and UV radicals are bombarded on the carbon surface, causing the phenomenon of decomposition and oxidation on the materials. Consequently, functional groups of oxygen are created on the carbon surface, inducing physical/chemical change on the surface of carbon-based papers.^37,38^

### Assembling of Flexible Allotropic Carbon-Paper Electrodes

Allotropic carbon-paper electrodes were prepared by aligning and assembling the different carbon papers over a supporting plastic sheet with a double-sided adhesive tape. The assembled carbon-paper/plastic sheet was then cut into electrode strips with a dimension of 30 mm length × 5 mm width using an electronic cutting machine (ScanNcut CM900, Brother Industries, Japan), followed by the encapsulation of electrode strips with an insulating adhesive tape (∼20 mm length). An active carbon working electrode was exposed with a surface area of 5 × 5 mm^2^, as shown in Figure 2a.

### Preparation of Prussian Blue-Functionalized Allotropic Carbon-Paper Electrodes

Prussian blue (PB) was deposited onto the allotropic carbon-paper electrodes by applying a constant potential at 0.4 V for 300 s in 1 M KCl and 3 mM HCl containing 1 mM K_3_(Fe(CN)_6_) and 1 mM FeCl_3_. Then, the PB-modified carbon-based electrodes were activated in a 0.1 M KCl and 0.01 M HCl solution by cycling at the potential range between −0.2 and 0.6 V with a scan rate of 0.05 V s^–1^ for 10 cycles. The PB-modified carbon-based electrodes were kept in a dark location at room temperature before being used.

### Preparation of PEDOT-Functionalized Allotropic Carbon-Paper Electrodes

PEDOT was deposited onto the allotropic carbon-paper electrodes by dynamic potential cycling between 0 and 1.2 V with a scan rate of 0.1 V s^–1^ in a solution containing 10 mM PEDOT and 2 mg mL^–1^ PSS for 20 cycles. Then, the PEDOT-modified carbon-based electrodes were activated in a 0.1 M KCl solution by dynamic potential cycling between 0 and 0.5 V with a scan rate of 0.1 V s^–1^ for 10 cycles.

### Characterizations and Electrochemical Measurements

The electrode morphology was characterized by a scanning electron microscope (SEM, LEO 155 Gemini, Zeiss, Germany). The element composition and mapping were determined by energy-dispersive X-ray spectroscopy (EDS, Oxford Instruments). Raman spectra were acquired with a LabRAM HR 800 Raman spectrometer (Horiba Jobin Yvon, France) using a 660 nm laser with a power of 5 mW.

All of the electrochemical measurements were performed by a BiPotentiostat/galvanostat μStat400 (Metrohm DropSens, S.L., Spain) at room temperature in an electrochemical cell consisting of a platinum wire, a silver/silver chloride (Ag/AgCl) electrode, and carbon paper as an auxiliary electrode, a reference electrode, and a working electrode, respectively. Electrochemical impedance spectroscopy was performed in 0.1 M KCl solution containing 5 mM [FeCN_6_]^3–/4–^ over the frequency range of 100 kHz–0.01 Hz with a voltage amplitude of 5 mV. The impedance spectra were then analyzed with ZSimpWin Software (AMETEK Scientific instruments).

## Results and Discussion

### Characterizations of the Bare 2D-, Nano-, and Microstructured Allotropic Carbon-Based Conducting Paper Substrates

**Figure 1:**
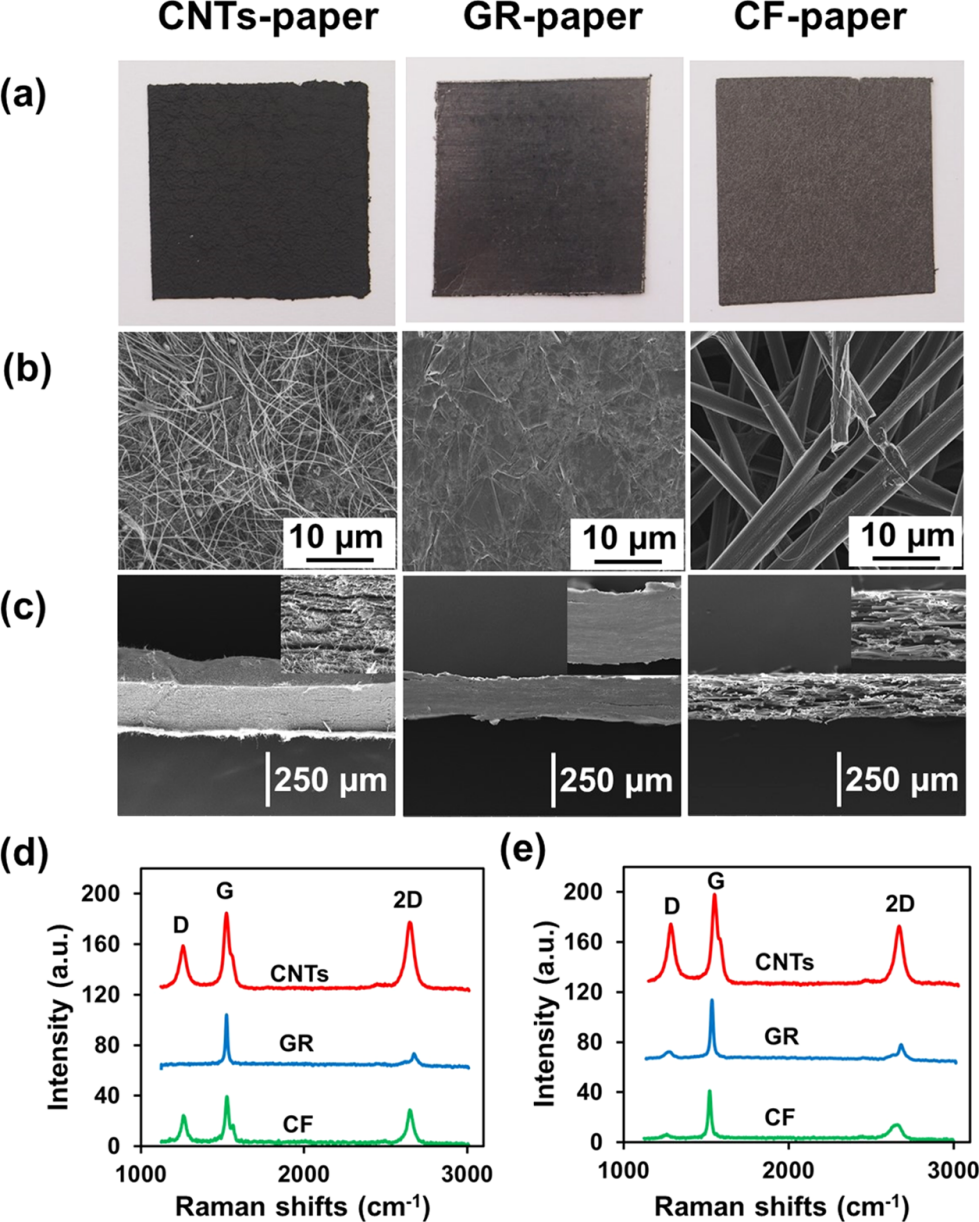
(a) Photographs of CNTs-paper, GR-paper, and CF-paper. (b) Top view and (c) cross-sectional view of SEM images for CNTs-paper, GR-paper, and CF-papers. (c) Inset shows high magnification. (d) Raman spectra of original allotropic carbon papers CNTs-paper (red line), GR-paper (blue line), and CF-paper (green line). (e) Raman spectra of allotropic carbon papers CNTs-paper (red line), GR-paper (blue line), and CF-paper (green line) after pretreatment with isopropanol, plasma, and isopropanol, respectively.

The physical appearance of different allotropic carbon papers is shown in Figure 1a. All of the carbon papers appear as thin paperlike sheets with a fairly homogeneous flat surface. Figure 1b,c shows the top view and cross-sectional view of scanning electron microscopy (SEM) images for different bare allotropic carbon papers including CNTs-paper, GR-paper, and CF-paper. It shows that the micro-/nanofeatures of the CNTs, GR, and CF are retained after the allotropic carbon is compressed into paper sheets. For CNTs-paper, the surface morphology appears as an interconnected CNT network forming a porous film structure and the cross-section image shows a multilayer structure of assembled CNTs. For GR-paper, the surface morphology is smooth and compact, which is composed of multilayer stacking of GR nanosheets, while the cross-sectional image shows some pieces of delaminated GR nanosheets. For CF-paper, it is composed of horizontally arranged microscale CF with large gaps between individual fibers forming a relatively loose film structure, and the cross-sectional image showed a multilayer structure of CF. In addition, the thickness of allotropic carbon-papers was measured by cross-section SEM image (Figure 1c). The thicknesses of CNTs-paper, GR-paper, and CF-papers were 356 ± 6, 195 ± 9, and 188 ± 2 μm, respectively. The structural properties for different bare allotropic carbon papers were further investigated by Raman spectroscopy. As shown in Figure 1d, all of the Raman spectra for bare CNTs-paper, GR-paper, and CF-paper exhibit two main characteristic bands that are specific for graphite-like materials with sp^2^ carbon, including the G band (∼1580 cm^–1^) and the 2D band (∼2670 cm^–1^) corresponding to the crystalline ordering of the graphitic basal plane and the stacking order, respectively.^39,40^ In addition, the CNTs-paper and CF-paper also possess another band located at ∼1330 cm^–1^ (D band), which is ascribed to the structural disorder and defect or the amorphous carbon material.^41^ The peak position, the intensity ratio of the D to G band (*I*_D_/*I*_G_), and the full width at half-maximum (FWHM) for all of the original allotropic carbon papers are summarized in Table 1. The *I*_D_/*I*_G_ ratio is commonly used for qualitative evaluation of the carbon material.^40^ However, the magnitude of FWHM is related to the orderliness of carbon materials.^42^ For GR-paper, the absence of the D band and the existence of a sharp G band (FWHM = 13.8 cm^–1^) indicate that a good graphene structure remained with symmetric bond stretching of all carbon–carbon bonds (sp^2^) in the honeycomb lattice structure.^41,43^ Moreover, the CNTs and CF-papers exhibit a high defect order with *I*_D_/*I*_G_ ratios of 0.542 and 0.564, respectively, implying a higher disorder as well as defects or impurities for CNTs-paper and CF-paper.

**Table 1:** Raman Spectroscopy Parameters of Allotropic Carbon-Based Conducting Papers before and after Pretreatment

		Raman shift/cm^–1^				
electrode materials	treatment	D band	G band	2D band	ratio of *I*_D_/*I*_G_	FWHM of G band/cm^–1^
CNTs-paper	before treatment	1330	1584	2654	0.54	37.4
GR-paper			1582	2681		13.8
CF-paper		1330	1585	2655	0.56	24.2
CNTs-paper	after treatment	1330	1584	2656	0.64	40.4
GR-paper		1341	1583	2681	0.10	14.0
CF-paper		1332	1581	2676	0.09	18.2

### Effect on Pretreatment and Electrochemical Properties of the 2D-, Nano-, and Microstructured Allotropic Carbon-Based Paper Electrodes

Physical (oxygen plasma)^37,38^ and chemical solvents such as nitric acid,^44^ potassium hydroxide,^45^ and isopropanol^46^ are commonly used for pretreatment and activation of electrode materials. For chemical pretreatment, nitric acid and potassium hydroxide act as a strong acid/base, and therefore, isopropanol was used in our study. The electrochemical properties of allotropic carbon-based paper electrodes before and after pretreatments were evaluated by cyclic voltammetry (CV) in 0.1 M KCl with 2 mM [FeCN_6_]^3–/4–^ as the redox probe (Figure S1). For CNTs-paper, the chemical isopropanol pretreatment could realize not only the decrease of background current and elimination of noise peak originating from the impurities during manufacture but also the improvement of electron transfer with a decreased peak-to-peak potential separation (Δ*E*_p_) compared to that of nontreated and plasma-treated CNTs-paper. Typically during the CNTs-paper construction, surfactants such as sodium dodecyl sulfate (SDS) or Triton X-100 are added to increase the ability of CNT dispersion, resulting in hindering the electrical conductivity. Isopropanol has the ability of removing residual surfactants that are retained on the surface of the carbon-paper material during the fabrication process.^31^ For GR-paper, the plasma treatment achieved the best electrochemical performance for [Fe(CN)_6_]^3–/4–^ with a sharper redox due to the introduction of oxygen-rich functional groups with enhanced electrode activities and increased surface hydrophilicity^47^ (Figure S2). On the contrary, there is no significant contribution from the pretreatment for CF-paper. Therefore, isopropanol treatment, plasma treatment, and nontreatment were chosen as optimized pretreatment conditions for CNTs-paper, GR-paper, and CF-paper, respectively, for further experiments and referred to as pretreated carbon papers in the following sections.

The morphology of carbon-based paper electrodes after pretreatment (isopropanol pretreatment for CNTs-paper and CF-paper, plasma pretreatment for GR-paper) was examined using SEM. Based on the SEM images, the morphologies of all of the carbon-based paper electrodes after pretreatment were similar to those before pretreatment (Figure S3). Therefore, pretreatment had no effect on the morphology of carbon-based paper electrodes.

Raman spectroscopy was used to evaluate the pretreatment effect with the optimized pretreatment conditions for the allotropic carbon papers (Figure 1e). All of the allotropic carbon-based papers show typical characteristic D-bands, G-bands, and 2D bands similar to laser-induced graphene (LIG) and GR materials as reported^48−50^ (Table S1). After treatment with isopropanol, the *I*_D_/*I*_G_ ratio of CNTs-paper increased from 0.54 to 0.64 and the FWHM value of the G band increased from 37.4 to 40.4, indicating the increase of structural defects on the surface of CNTs-paper (Table 1). Such structural defects on CNTs are favorable for the enhanced electrochemical reaction activity,^51^ which is consistent with the improved electron-transfer effect in CV (Figure S1c). In the case of GR-paper, the new appearance of the D band after plasma treatment at 1341 cm^–1^ and the slight increase of the FWHM value of G from 13.8 to 14.0 can be ascribed to the formation of disorder at graphene due to oxygenated functional groups,^52^ which was further certified by the improved hydrophilicity with the decreased water contact angle from 90.3 to 73.1° (Figure S2). Such a disorder in graphene is favorable for the improved electrochemical reaction activity,^52,53^ which can be clearly seen from the sharper redox peaks for GR-paper treated with plasma (Figure S1b). On the other hand, a decreased intensity of characteristic peaks was observed in the case of CF-paper after isopropanol pretreatment. The *I*_D_/*I*_G_ ratio decreasing from 0.56 to 0.09 corresponds to an increase in the graphitic crystalline structure over the disorder.^54^

**Figure 2:**
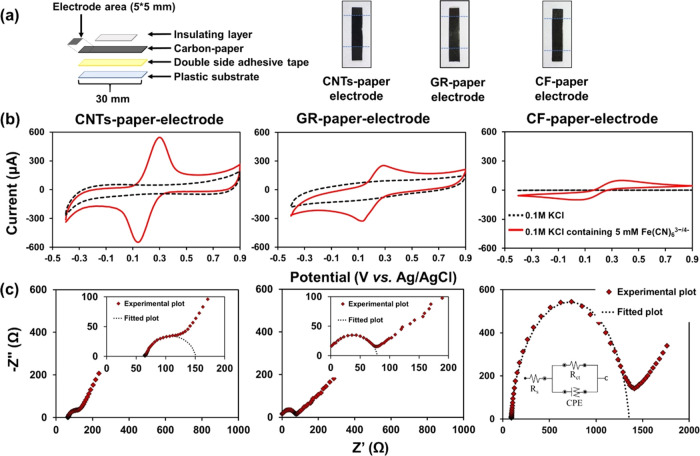
(a) Schematic diagram for the assembling of flexible carbon-paper electrodes and corresponding digital images. (b) Cyclic voltammograms and (c) Nyquist plots of CNTs-paper, GR-paper, and CF-paper in 0.1 M KCl containing 5 mM [FeCN_6_]^3–/4–^.

The allotropic carbon-paper-based flexible electrodes were prepared via shaping of the conducting papers into strips followed by assembling the carbon-paper strip with an insulating substrate and an encapsulation layer using adhesive tape (Figure 2a). The electrochemical properties of the resulting carbon-paper electrodes were characterized by CV in 0.1 M KCl containing 5 mM [FeCN_6_]^3–/4–^. As shown in Figure 2b, all allotropic carbon-paper-based electrodes demonstrated a pair of reversible redox peaks, revealing a good reversible electrochemical process at the electrode interface. The anodic peak currents for the CNT-, GR-, and CF-paper-based electrodes were 460, 180, and 96 μA, respectively, in which the anodic peak currents of CNTs-paper were 2.6 and 4.8 times higher than those of GR- and CF-paper-based electrodes. Such an increase in the peak current is likely due to the porous and nanostructured CNT network possessing a higher active electrode surface area. Besides, the CNTs and GR-paper-based electrodes displayed relatively smaller Δ*E*_p_ values of 160 and 150 mV, respectively, while the CF-paper-based electrode exhibited the largest Δ*E*_p_ value of 330 mV. Moreover, electrochemical kinetics of carbon paper were further investigated by studying the effect of scan rate. The current response increased linearly versus the square root of the scan rate from 0.01 to 0.17 V s^–1^ (Figure S4), suggesting that the electrochemical kinetic process is diffusion-controlled. The apparent diffusion coefficients of the redox probe toward the allotropic carbon-paper-based electrodes were calculated to be 7.53 × 10^–5^ (CNTs-paper), 3.38 × 10^–6^ (GR-paper), and 3.53 × 10^–6^ (CF-paper) cm2 s^–1^ using the Randles Sevcik equation. Furthermore, CNTs-paper showed better electrochemical characteristics compared to GR-paper due to the interconnected CNT nanofibers with a porous electrode morphology compared with GR-paper with a compacted stacking of the GR nanosheet.

EIS was performed to examine the electron-transfer kinetics of the bare carbon-paper electrodes in the frequency range from 0.01 Hz to 100 kHz in 0.1 M KCl containing 5 mM [FeCN_6_]^3–/4–^. Figure 2c shows the corresponding Nyquist plots and the EIS fitting curve for the CNT-, GR-, and CF-paper-based flexible electrodes. The diameter of the semicircle in the fitted curves was used to measure the charge-transfer resistance (*R*_ct_). The *R*_ct_ is used to characterize the kinetics of the redox probe on the surface of the electrode material.^55^ The *R*_ct_ values for electrodes based on CNTs-paper (87.77 Ω) and GR-paper (87.32 Ω) were much smaller than that of the CF-paper-based electrode (1,259 Ω). It is worthy to note that the *R*_ct_ value of the nanostructured carbon papers (i.e., CNTs and GR) was much lower than that of the microstructured carbon-paper (i.e., CF) electrode, which is in agreement with the corresponding electrochemical characteristics measured from CV. Moreover, the similar *R*_ct_ values for CNTs-paper and GR-paper can be ascribed to their intrinsic nanostructure property.

Taking the diffusion process into consideration, the heterogeneous electron-transfer-rate constant (*k*°) was estimated via EIS for the allotropic carbon materials as the following equation^56^
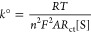
1where *k*° is the heterogeneous electron-transfer rate constant, *A* is the geometric area of the working electrode (cm^2^), *T* is the temperature (K), *n* is the number of electron transfers per molecule of the redox probe, *R* is the universal gas constant, [S] is the bulk concentration of the redox probe (mol cm^–3^), and *R*_ct_ is the charge-transfer resistance. The *k*° values for CNTs-, GR-, and CF-paper-based electrodes were calculated to be 2.43 × 10^–3^, 2.44 × 10^–3^, and 1.69 × 10^–4^ cm s^–1^, respectively. The *k*° values of CNTs- and GR-paper-based electrodes were ∼14 times higher than that of the CF-paper-based electrode. It indicates that the nanostructures of CNTs and the uniform packing of GR sheets within the assembled bulk conducting papers preserve the high active surface area and thus promote faster electron-transfer kinetics compared to the CF-paper.

### Bending Stability of the 2D-, Nano-, and Microstructured Carbon-Based Flexible Electrodes

**Figure 3:**
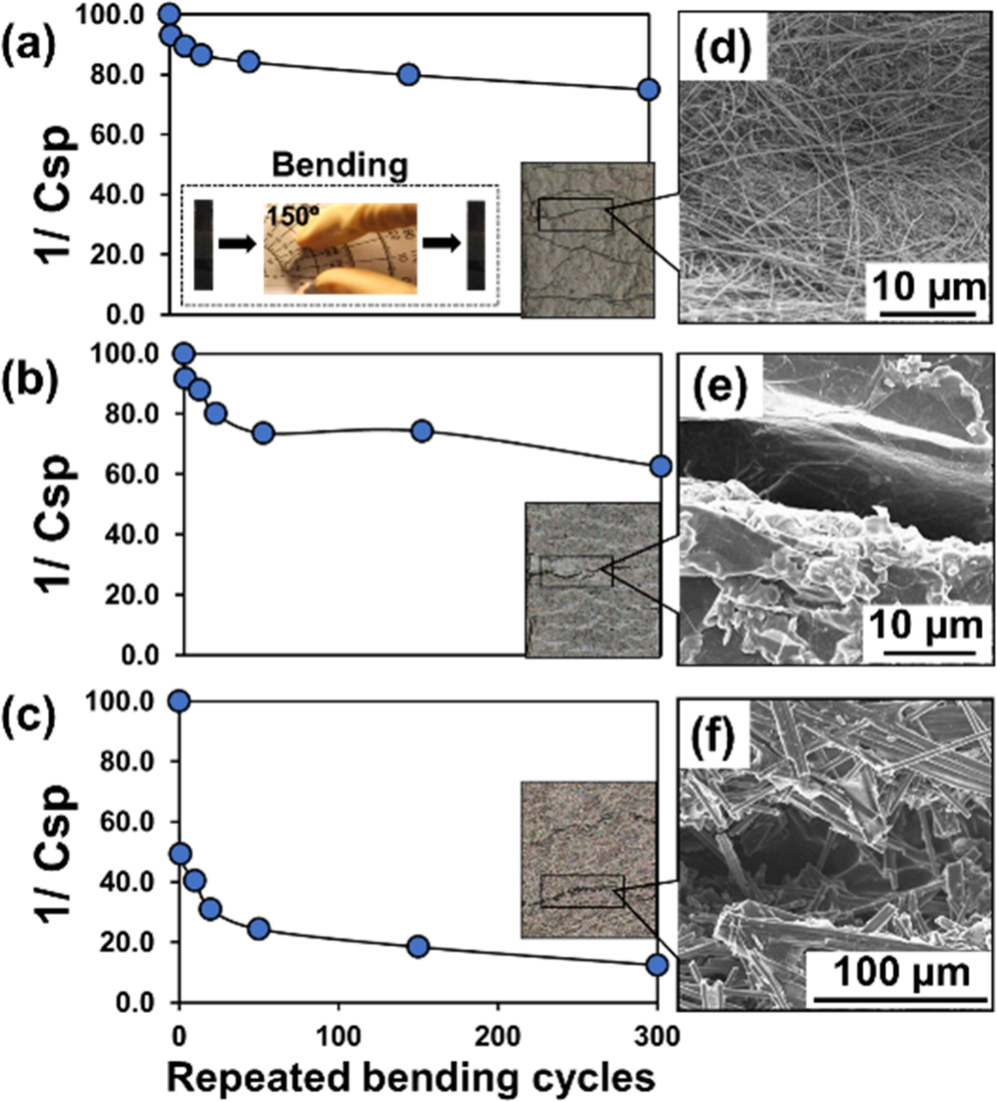
Bending stability test evaluated by 1/*C*_sp_ with repeated bending cycles and corresponding SEM images in high magnification after 300 cycles bending for (a) CNTs-paper-, (b) GR-paper-, and (c) CF-paper-based flexible electrodes. (a) Inset shows the bending angle. SEM image in low magnification at bending position for (d) CNTs-paper, (e) GR-paper, and (f) CF-paper after repeated 300 bending cycles.

To investigate the mechanical properties of different allotropic carbon papers as flexible electrodes, the CNTs-, GR-, and CF-paper electrodes were subjected to repeated bending cycles at a 150° bending angle for 300 cycles (Figure 3). The bending stability was evaluated based on the hypothesis that structural fracture will create cracks or defects on the surface of the allotropic carbon-based electrodes and consequently increase the appearance of the surface area of the electrodes. The increase in the electrode surface area would enhance ion interaction at the electrode interface, leading to an increase in the area-specific capacitance. The changes in capacitance were evaluated by CV scanning at a potential ranging from −0.2 to 0.6 V at 0.05 V s^–1^ in 0.1 M KCl solution. The specific capacitance was calculated from CVs according to eq 2.^57,58^
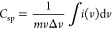
2where *C*_sp_ is the specific capacitance, *m* is the geometric area of the working electrode (cm^2^), *v* is the scan rate, Δ*v* is the potential window, and ∫*i*(*v*)d*v* is the area under the CV curve. The apparent bending stability caused by the cracks and defects is thus inversely proportional to the change in capacitance, and therefore can be defined as 1/*C*_sp_. The apparent bending stability of different allotropic carbon-based electrodes as a function of bending cycles is shown in Figure 3a–c, whereas the initial capacitance before bending was normalized as 100%. The result shows that after an initial 10 bending cycles, the 1/*C*_sp_ values of CNTs-, GR-, and CF-paper electrodes dropped to 89.4, 88.0, and 40.5% (i.e., decreases of 10.6, 12.0, and 59.5%), respectively. The obtained bending stabilities of the CNTs-paper and GR-paper electrodes were 5.61 and 4.96 times better in comparison to the CF-paper. However, after 20 bending cycles, the 1/*C*_sp_ values of CNTs-paper, GR-paper, and CF-papers showed smaller decreases of 2.9, 7.9, and 9.6%, respectively. Interestingly, after a high number of continuous bending for 150 cycles, the impact on 1/*C*_sp_ values of the CNTs-paper, GR-paper, and CF-paper resulting from the bending is significantly further reduced with only small decreases of 6.6, 5.8, and 12.4%, respectively. According to the thickness measurements by SEM (Figure 1c), despite the CF-paper being the thinnest carbon substrate, it has the lowest bending stability compared with CNTs- and GR-paper. However, carbon papers composed of nanostructured materials provide better bending stability, which is likely due to the nanodimensional feature of the assembled CNTs and GR being more resistant to structural fracture upon bending. The SEM images of the CNTs-, GR-, and CF-paper electrodes after 300 bending cycles are shown in Figure 3d–f. The surface of the CNTs-paper showed a few wrinkles and the GR-paper showed creases, while the CF-paper had a fractured and broken fiber structure around the bending site. The morphologies of the bent carbon papers agreed with the corresponding results obtained from the 1/*C*_sp_ measurement. More interestingly, we observed that the initial bending cycles created a larger impact on the stability of all of the carbon papers, followed by a relatively more stable value after the initial bending cycles. This observation could be explained by the classical properties and characteristics of paper substrates—that defects were created upon initial bending while remaining fairly stable upon rebending. The magnitude of the defects associated with the bending stability is highly related to the structure and organization of the allotropic carbon materials’ packed-paper format. Additionally, the bending stability of different allotropic carbon papers was evaluated by CV using a [FeCN_6_]^3–/4–^ redox probe (Figure S5). After 300 bending cycles, the oxidation peak currents measured by the CNTs-, GR-, and CF-papers were increased by 42.8, 45.7, and 94.5%, respectively. The increased peak current is likely due to the cracks or defects on the surface of the allotropic carbon-based electrodes with an increased appearance of the surface area of the electrodes. Both capacitive and charge-transfer characterizations indicate that the CNTs-paper and GR-paper electrodes have a better bending stability compared with the CF-paper electrode.

### PB-Functionalized Allotropic Carbon-Paper Electrodes (PB-Carbon-Paper) for Electrochemical Sensing

**Figure 4:**
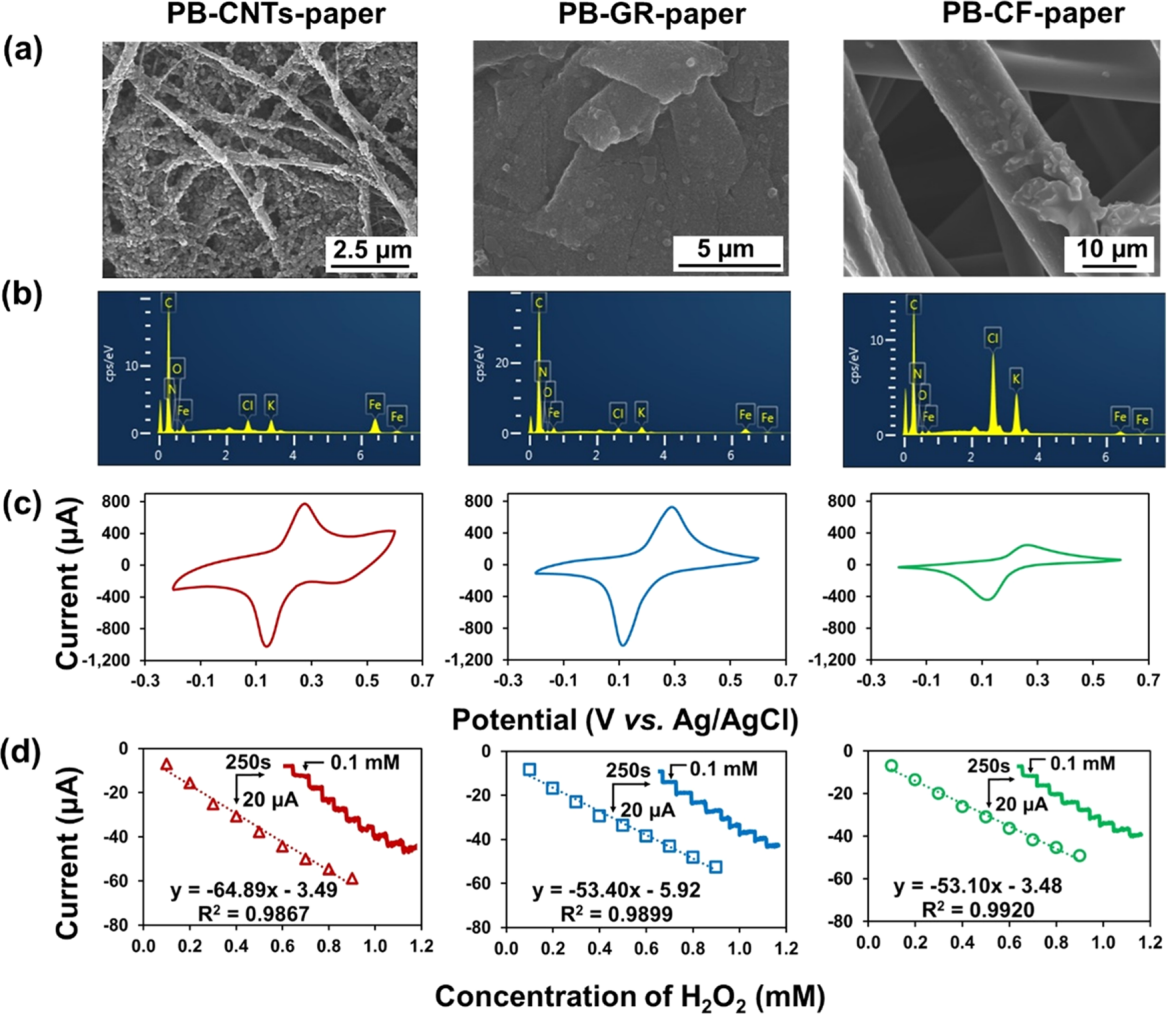
(a) SEM images and (b) EDX spectra of PB-CNTs-paper, PB-GR-paper, and PB-CF-paper. (c) Cyclic voltammograms of PB-CNTs-paper, PB-GR-paper, and PB-CF-paper in 0.1 M KCl solution containing 0.01 M HCl. (d) Calibration curves of electrochemical sensing of H_2_O_2_ at PB-CNTs-paper, PB-GR-paper, and PB-CF-paper; insets show the corresponding current–time curve at a working potential of 0.0 V in 0.1 M PBS (pH 6.0).

For advanced electronic devices, modification of the electrodes with inorganic catalysts or organic conducting polymers is essential. Carbon-based papers were activated by appropriate pretreatment before being functionalized with inorganic catalysts or organic conducting polymers. To explore the postmodification performance of conducting carbon papers for electrochemical sensing application, a commonly used inorganic catalyst PB was electrochemically deposited onto different carbon-paper electrodes for the preparation of PB-carbon-paper electrodes for hydrogen peroxide (H_2_O_2_) detection, which is an important analyte in the biological system that is generated from reactions catalyzed by oxidases.^59^ The morphology and chemical characteristics of the PB-modified carbon-paper electrodes were evaluated by SEM and energy-dispersive X-ray analysis (EDX). The SEM images showed the deposition of granulated PB particles onto CNTs-paper, GR-paper, and CF-paper (Figure 4a). The EDX spectra illustrate the presence of the characteristic elements, i.e., iron (Fe) and nitrogen (N), originating from the PB, indicating the successful preparation of PB-CNTs-paper, PB-GR-paper, and PB-CF-paper electrodes (Figure 4b). The electrochemical properties of the PB-modified carbon-paper electrodes were characterized by CV showing a typical pair of quasi-reversible peaks resulting from the redox behavior of Fe^3+^/Fe^2+^ in PB (Figure 4c). The CVs of the PB-CNTs-, PB-GR-, and PB-CF-papers showed a pair of characteristic peaks of PB.^60^ The cathodic peak currents of the PB-CNTs- and PB-GR-paper electrodes were higher (∼1.8 and ∼2.2 times) compared with that of the PB-CF-paper electrode. This indicates that a higher amount of PB deposited onto the CNTs- and GR-paper electrodes related to the relatively dense electrode surface morphology compared with the CF-paper electrode, as shown in the SEM image (Figure 4a). Moreover, the PB-CNTs- and PB-GR-paper electrodes exhibit a significantly sharper redox peak compared with the PB-CF-paper electrode. The sharpness of the PB redox peaks represents the inorganic polycrystalline structure on carbonaceous materials and was used to evaluate the quality of PB.^61,62^Figure 4d (insets) shows the current–time responses measured at 0 V with the successive addition of H_2_O_2_ for PB-CNTs-, PB-GR-, and PB-CF-paper electrodes with a fast response time that reaches a steady-state current within 10–20 s. All of the PB-modified carbon-paper electrodes exhibited a good linear relationship in the concentration range of 0.1–0.9 mM with a correlation coefficient>0.99 (Figure 4d). Among all, the PB-CNTs-paper electrode showed the highest sensitivity of 64.89 μA mM^–1^, while the PB-GR-paper and PB-CF-paper electrodes also presented good sensitivities of 53.40 and 53.10 μA mM^–1^, respectively. The assembled CNT-paper electrode with an interconnected nanofibrous structure with a porous electrode morphology compared with the compacted GR-paper electrode (Figure 4a) with a large appearance of surface area facilitates the deposition of PB, resulting in better electrochemical and sensing performances. The analytical performances of the PB-modified carbon-paper electrodes for H_2_O_2_ detection have been compared with other reports, as shown in Table S2. Compared to other reports, the PB-modified carbon-based paper exhibited a high sensitivity with low potential detection. As such, the carbon-based papers could be an alternative substrate for the application of sensors/biosensors.

### PEDOT-Functionalized Allotropic Carbon-Paper Electrodes (PEDOT-Carbon-Paper) for Charge Storage

**Figure 5:**
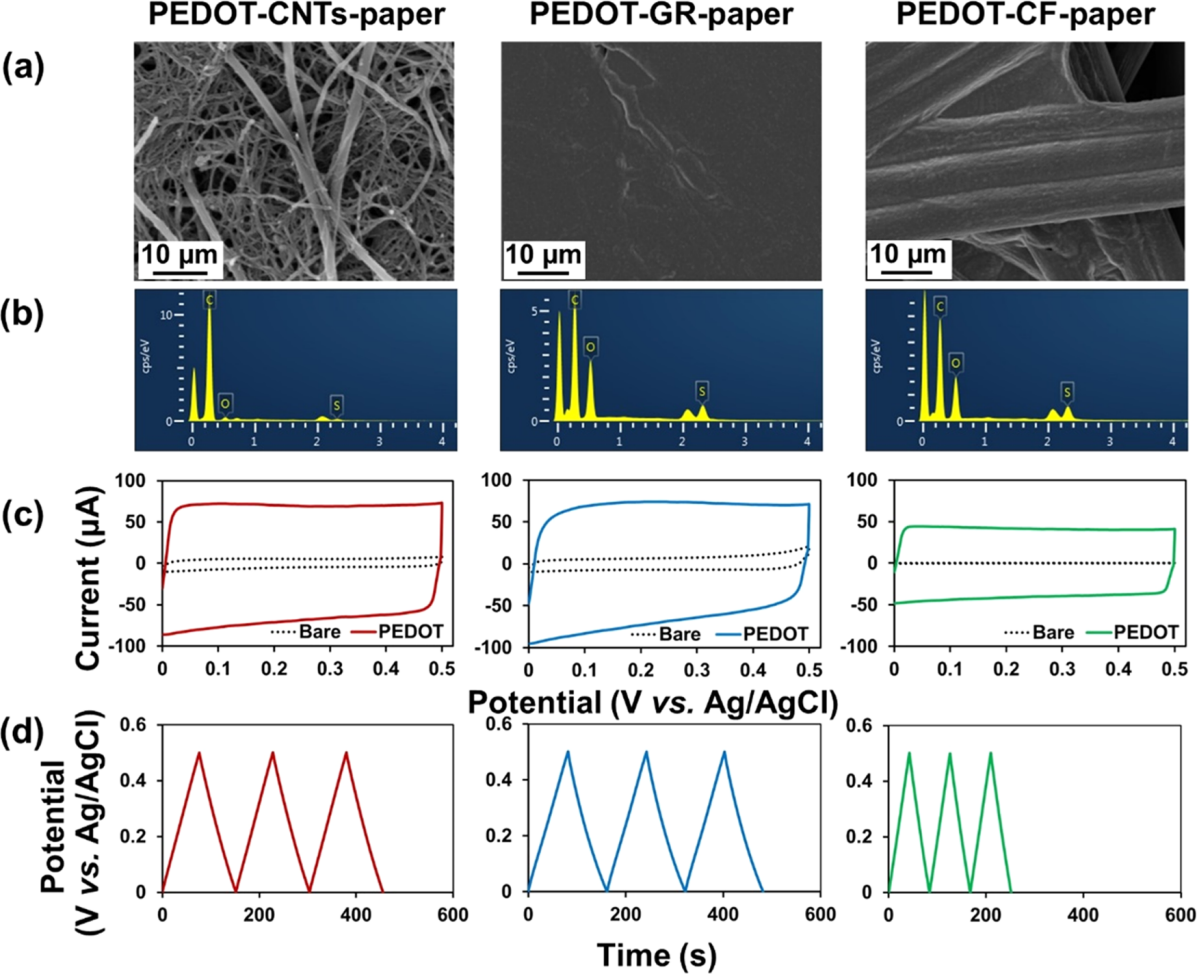
(a) SEM images and (b) EDX spectra of PEDOT-CNTs-paper, PEDOT-GR-paper, and PEDOT-CF-paper. (c) Cyclic voltammograms of CNTs-paper, GR-paper, and CF-paper in 0.1 M KCl solution before and after modification of PEDOT at a scan rate of 0.05 V s^–1^. (d) Galvanostatic charge–discharge curves of PEDOT-CNTs-paper, PEDOT-GR-paper, and PEDOT-CF-paper in 0.1 M KCl solution.

To evaluate the electrochemical energy storage applications, supercapacitive electrodes were fabricated by electrodeposition of PEDOT onto different allotropic carbon-paper electrodes, in which PEDOT is a pseudocapacitive material having a high specific capacitance and ionic conductivity.^63,64^ The morphology and chemical characteristic of the PEDOT-carbon-paper electrode were characterized by SEM and EDX analyses. A PEDOT film was electrochemically deposited onto the CNTs-, GR-, and CF-paper electrodes (Figure 5a). The EDX spectra showed that the typical sulfur (S) and oxygen (O) elements originating from the PEDOT indicate the successful preparation of PEDOT-CNTs-paper, PEDOT-GR-paper, and PEDOT-CF-paper electrodes (Figure 5b). The electrocapacitive performance of the PEDOT-carbon-paper electrodes was evaluated with cyclic voltammetry (CV) and galvanostatic charge–discharge (GCD) measurement in 0.1 M KCl solution. Figure 5c shows the typical symmetric rectangular-shaped CV curves resulting from the electrochemical pseudocapacitive properties of PEDOT. The GCD curves (Figure 5d) obtained from all of the PEDOT-carbon-paper electrodes showed a symmetric triangular-shaped curve illustrating the typical electrocapacitive behavior of the electrodes. The PEDOT-CNTs-paper electrode and the PEDOT-GR-paper electrode showed slower discharge kinetics (i.e., longer times of charge or discharge) in comparison with the PEDOT-CF-paper electrode with the same geometric electrode surface area, which is likely contributed by the nanostructural feature of the CNTs and GR with a higher surface area leading to a longer charging/discharging time. The specific capacitance (*C*_sp_) representing the charge storage ability of the PEDOT-modified carbon-paper electrodes was calculated from discharge slopes at various current densities according to eq 3 ^65^
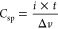
3where *i* is the current density (A cm^–2^), *t* is the charge/discharge time (s), and Δ*v* is the electrochemical potential window (V). The calculated *C*_sp_ values for the PEDOT-CNTs-paper, PEDOT-GR-paper, and PEDOT-CF-paper electrodes were 12.34, 12.49, and 6.86 mF cm^–2^, respectively. This result is consistent with the CV measurement showing a similar trend on the electrochemical pseudocapacitive behavior related to the size of the rectangular-shaped CV curves. This result demonstrates the effective modification of all carbon-paper electrodes with PEDOT for charge storage. However, the PEDOT-CNTs-paper and PEDOT-GR-paper composed of nanostructured carbon materials showed a significantly higher *C*_sp_ compared with the PEDOT-CF-paper electrode. The capacitance values of the PEDOT-functionalized carbon-based paper electrodes were compared with the literature-reported PEDOT-modified electrodes, showing comparable and in some cases higher capacitance values (Table S3), likely attributed to the intrinsic nano-/microstructure within the allotrope carbon-paper substrates with a higher active surface area.

### Physicoelectrochemical Properties of 2D-, Nano-, and Microstructured Carbon-Based Paper as Flexible Electrodes

The evaluation of the physicoelectrochemical properties for various allotropic carbon papers as flexible electrodes is summarized in Table 2. The nano and micro features of the CNTs, GR, and CF were retained after the allotropic carbon was compressed into paper sheets. All allotropic carbon papers show a good characteristic G band corresponding to the in-plane vibrations of the SP_2_ bonded carbon, which is essential as a conducting electrode substrate, while structural defects for the CNTs and GR-papers characterized by the D band could be enhanced via physical or chemical treatment to deliver an improved electrochemical reaction activity at the electrode interfaces. The nanostructured CNTs- and GR-paper electrodes delivered faster electrode kinetics, low *R*_ct_, and high *k*° values compared with the microstructured CF-paper. Moreover, the bending stability of the CNTs- and GR-paper electrodes composed of nanostructured carbon showed a significantly higher stability facilitated by the nanodimensional feature of the assembled CNTs and GR for a better resistance on structural fracture upon bending. Despite the price of the CNTs- and GR-papers being higher compared with the CF-paper, their good electrochemical performance and high bending stability are essential for the development of advanced flexible and wearable electronics.

**Table 2:** Summary of the Evaluation on Physioelectrochemical Properties of Allotropic Carbon-Based Conducting Paper as Flexible Electrodes

parameters		CNTs-paper	GR-paper	CF-paper
structure properties	morphology	porous nanofibrous network	multilayer nanosheet assembly	porous microfibrous network
	carbon structure (G band)	good SP_2_ crystalline structure	excellent SP_2_ crystalline structure	good SP_2_ crystalline structure
	carbon structure (D band)	high defects	na	moderate defects
	activation	enhanced defects by isopropanol treatment	introduced defects by plasma treatment	no improvement
electrochemical properties	electrode kinetic, Δ*E*_p_ (mV)	fast (160 mV)	fast (150 mV)	slow (330 mV)
	charge-transfer resistance, *R*_ct_ (Ω)	small (87.77 Ω)	small (87.32 Ω)	large (1259 Ω)
	heterogeneous electron-transfer rate constant, *k*° (cm^–1^)	fast (2.43 × 10^–3^ cm^–1^)	fast (2.44 × 10^–3^ cm^–1^)	slow (1.69 × 10^–4^ cm^–1^)
bending stability	initial 20 bending cycles	good (86.5% retain)	good (80.1% retain)	poor (30.9% retain)
	from 20 to 300 cycles	excellent (75% retain)	good (62% retain)	poor (12.5% retain)

## Conclusions

The physicochemical properties and electrochemical performance for various allotropic carbon papers as flexible electrodes were studied. All allotropic carbon papers showed a good characteristic G band corresponding to the in-plane vibrations of SP_2_ bonded carbon, which is essential as a conducting electrode substrate, while structural defects for the CNTs- and GR-papers characterized by the D band could be enhanced via physical or chemical treatment to deliver an improved electrochemical reaction activity at the electrode interfaces. The nanostructured CNTs- and GR-paper electrodes delivered faster electrode kinetics, lower *R*_ct_, and higher *k*° values compared with the microstructured CF-paper. Moreover, the bending stabilities of the CNTs- and GR-paper electrodes composed of nanostructured carbon showed a significantly higher stability facilitated by the nanodimensional feature of the assembled CNTs and GR for a better resistance on structural fracture upon bending. We further demonstrated the functionalization of the allotropic carbon papers with an inorganic catalyst to fabricate PB-carbon-paper for electrochemical sensing and an organic conducting polymer to fabricate PEDOT-carbon-paper for energy storage. Carbon-based conducting papers provide a new route for the design and fabrication of advanced flexible electronic devices. Our studies provide comprehensive studies on the fundamental physioelectrochemical characteristics of allotropic nano-/microstructured carbon papers, which is critical for the future development of advanced paper-based flexible electronic devices.

## Supporting Information Available

Details of the pretreatment effect using CV and SEM of allotropic carbon papers, contact-angle measurement, effect of scan rate; table showing the Raman spectroscopy parameters of allotropic carbon-based papers compared with other carbon-based materials, and comparison of the performance of H_2_O_2_ sensors and specific capacitance of different PEDOT-modified electrodes (PDF)
