# Supplementary material for: Evaluation on the Intrinsic Physicoelectrochemical Attributes and Engineering of Micro-, Nano-, and 2D-Structured Allotropic Carbon-Based Papers for Flexible Electronics
Source: Langmuir. 2021 Dec 3;37(49):14302–13. doi: 10.1021/acs.langmuir.1c02121 (PMC8675137; doi:10.1021/acs.langmuir.1c02121)
Supplement: Supplementary file 1 — la1c02121_si_001.pdf [file la1c02121_si_001.pdf]

Supporting Information

Evaluation on the Intrinsic Physico-Electrochemical Attributes and Engineering of Micro, Nano, 2D-Structured Allotropic Carbon-based Papers for Flexible Electronics

Supatinee Kongkaew,[a,b,c,d] Lingyin Meng,[a] Warakorn Limbut,[b,c,e] Proespichaya Kanatharana,[b,c,d] Panote Thavarungkul,[b,c,d] Wing Cheung Mak [a*]

[a] *Biosensors and Bioelectronics Centre, Division of Sensor and Actuator Systems, Department of Physics, Chemistry and Biology, Linköping University, SE-581 83 Linköping, Sweden*

[b] *Center of Excellence for Trace Analysis and Biosensor, Prince of Songkla University, Hat Yai, Songkhla 90110, Thailand*

[c] *Center of Excellence for Innovation in Chemistry, Faculty of Science, Prince of Songkla University, Hat Yai, Songkhla 90110, Thailand*

[d] *Division of Physical Science, Faculty of Science, Prince of Songkla University, Hat Yai, Songkhla 90110, Thailand.*

[e] *Division of Health and Applied Sciences, Faculty of Science, Prince of Songkla University, Hat Yai, Songkhla 90110, Thailand.*

*Corresponding author: wing.cheung.mak@liu.se

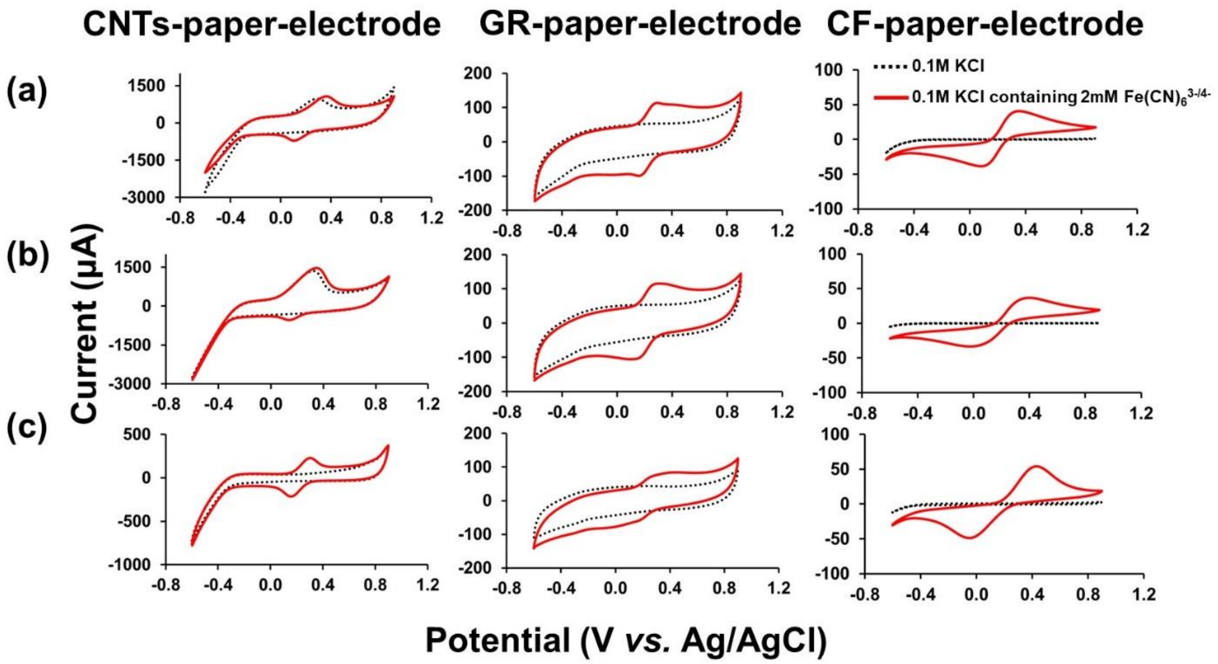

Figure S1. Effect of pre-treatments. Cyclic voltammograms of CNTs-paper, GR-paper, and CF-paper with different activation approaches including **(a)** non-treatment, **(b)** plasma treatment, and **(c)** chemical cleaning, respectively in 0.1 M KCl containing 2 mM $[FeCN_6]^{3-/4-}$.

The electrochemical performance of allotropic carbon paper based electrodes before and after pre-treatments was evaluated by cyclic voltammetry in 0.1 M KCl containing 2 mM of $[FeCN_6]^{3-/4-}$ with the scan rate of 0.05 V s^{-1}. The improvement of the electrochemical properties of carbon papers was evaluated by peak-to-peak potential separation ($\Delta E_p = E_{ox} - E_{re}$), and the ratio of redox peak currents for standard redox compounds (ideal value for a reversible redox process is $I_{pa}/I_{pc} \sim 1$). As shown in **Figure S1**, the improvement of electrochemical kinetic was clearly observed for CNTs-paper electrode after treatment with isopropanol, such as lower ΔEp (0.16 V) and lower background current compared to that of non-treated (ΔEp = 0.20 V) and plasma treated (ΔEp = 0.25 V) CNTs-paper electrodes and with a good I_{pa}/I_{pc} value of 0.94. Interestingly, there was an impurity oxidation peak for CNTs-paper at ~0.3 V scanned in pure electrolyte without redox probe (black line) and the impurity peak was removed after isopropanol pre-treatment (red line). GR-paper shows a sharper redox peak after plasma treatment with the improvement of I_{pa}/I_{pc} (1.02) compare with non-treated (I_{pa}/I_{pc} = 1.09) and isopropanol treated (I_{pa}/I_{pc} = 1.28) GR-paper electrode. In contrast, CF-paper electrodes treated with isopropanol or plasma treatment showed an

increased in the ΔE_p to 0.36 V and 0.46V, respectively compared with the non-treated CF-paper electrode. While no treatment exhibited the lowest ΔEp of 0.24 V with good I_{pa}/I_{pc} of 1.06.

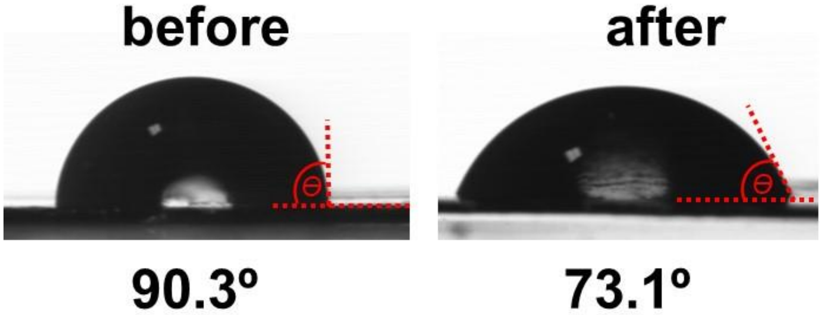

Figure S2. Contact angle measurement of GR-paper before and after plasma treatment**.**

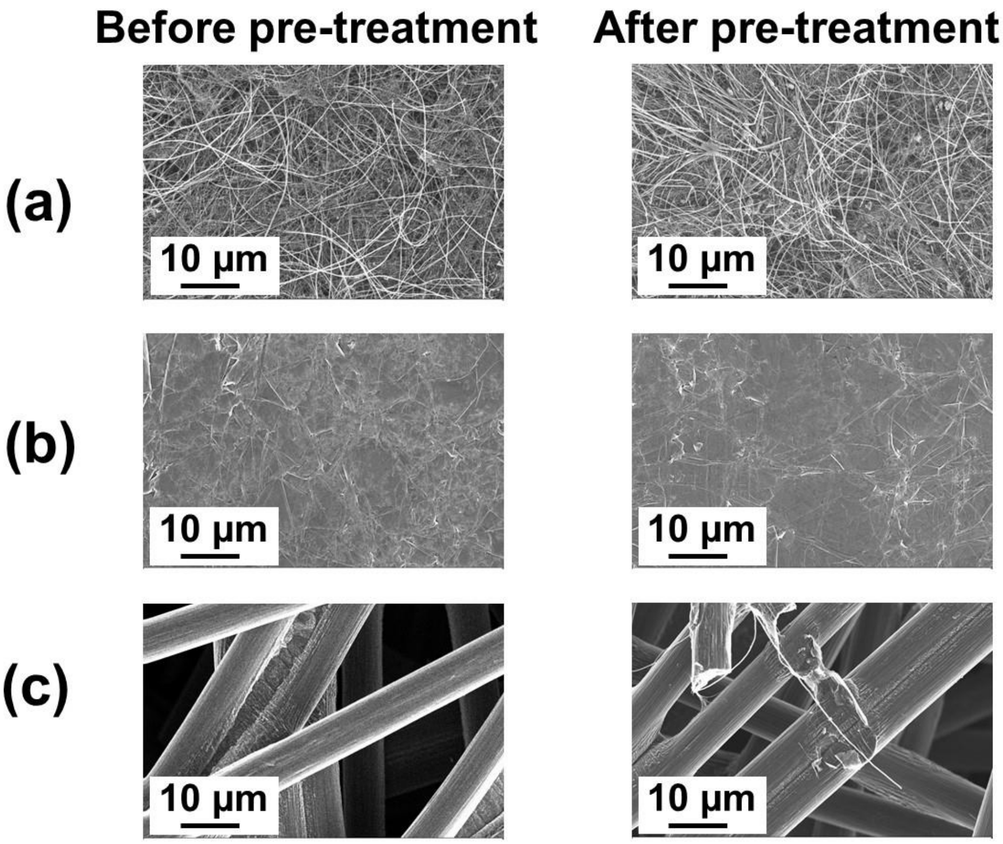

Figure S3. The morphology studied of (a) CNTs-paper, (b) GR-paper, (c) CF-paper before and after pre-treatment (isopropanol pre-treatment for CNTs-paper and CF-paper, and plasma pretreatment for GR-paper).

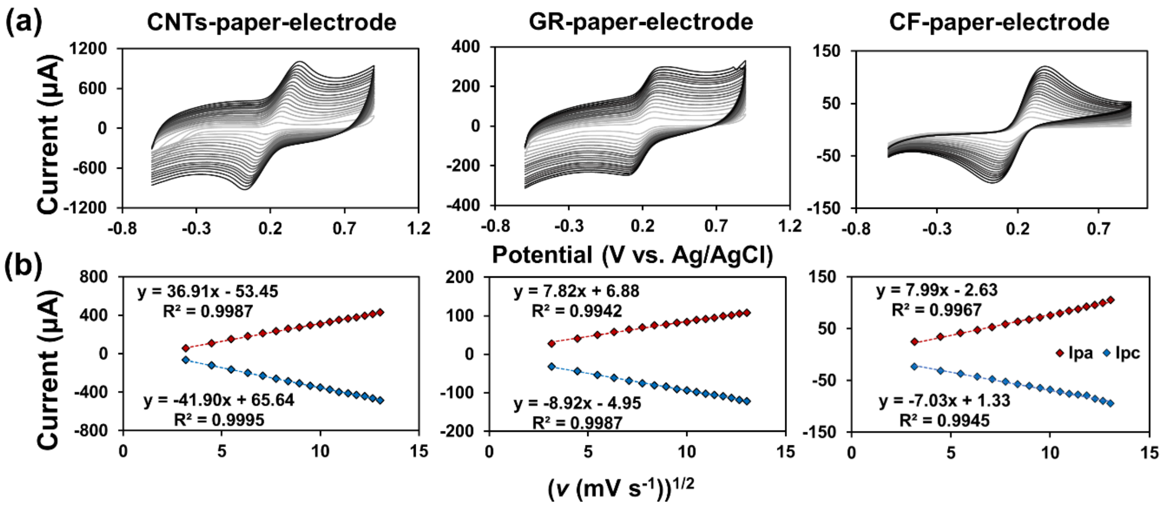

Figure S4. Effect of scan rate. **(a)** Cyclic voltammograms of CNTs-paper, GR-paper, and CF-paper-electrodes in 0.1 M KCl containing 2 mM $[FeCN_6]^{3-/4-}$ with various scan rate. **(b)** Plots of the cathodic and anodic peak current versus the square root of scan rates from 0.01-0.17 V s^{-1} for CNTs-paper, GR-paper, and CF-paper-electrodes.

The diffusion coefficient of the allotropic carbon paper electrodes was calculated by Randles Sevcik equation:

$$I_p = (2.69\times10^5)n^{3/2}FAD^{1/2}Cv^{1/2} \qquad \text{Eq.1}$$

where I_p is the peak current (A), n is the charge transfer number in the reaction redox couple, A is the geometric area of working electrode (0.25 cm^2), F is constant of Faraday (96,485 C mol^{-1}), C is concentration of redox prob (mol cm^{-3}), D is the diffusion coefficient (cm^2 s^{-1}), v is the scan rate (V s^{-1}). Based on the slope of linear equation, the diffusion coefficient was estimated to be 7.53×10^{-5}, 3.38×10^{-6}, 3.53×10^{-6} cm^2 s^{-1} for CNTs-paper, GR-paper and CF-paper, respectively.

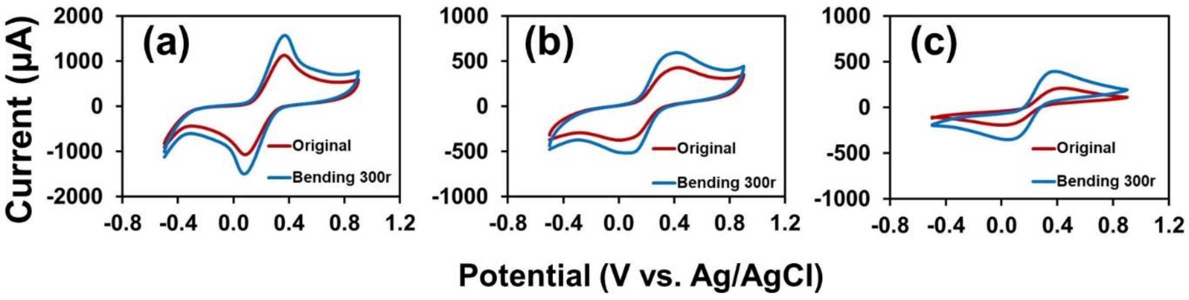

Figure S5. Bending stability test. Cyclic voltammograms of original **(a)** CNTs-paper, **(b)** GR-paper, and **(c)** CF-paper (red line) and after pre-treatment (blue line) with chemical cleaning, plasma treatment, and non-treatment, respectively in 0.1 M KCl containing 5 mM $[FeCN_6]^{3-/4-}$.

Table S1 Raman spectroscopy parameters of allotropic carbon-based papers comparison with other carbon-based materials.

**Electrode materials**	**Raman shift/ cm^{-1}**			**Ratio of I_D/I_G**	**FWHM of G band/ cm^{-1}**
	**D band**	**G band**	**2D band**		
CNTs-paper	1330/1330*	1584/1584*	2654/2656*	0.54/0.64*	37.4/40.4*
GR-paper	n.a./1341*	1582/1583*	2681/2681*	n.a./0.10*	13.8/14.0*
CF-paper	1330/1332*	1585/1581*	2655/2676*	0.56/0.09*	24.2/18.2*
LIG [1]	1330	1580	2700	0.64	n.a
3D-Gr [2]	n.a.	1579	2698	n.a.	15
Gr [3]	1365	1594	2727	1.3	68.1

Note; * means the value obtained after pre-treatment.

LIG : laser induced graphene

3D-Gr : three dimensional graphene

Gr : graphene

Table S2. Comparison of different Prussian blue modified electrodes for the determination of H_2O_2.

Modified electrodes	Potential (V)	Linear range (mM)	Sensitivity (μA/μM)	LOD (μM)	Ref.
PB/PET[a]	-0.10	0.005-0.5	0.0112	1.9	4
PB/3DGrE[b]	-0.15	0.001-0.7	0.0407	0.11	5
PBNPs/SPEs[c]	0.0	0-4.5	0.053	0.2	6
PB/SPCE[d]	0.15	0.02-0.7	0.0000466	20	7
PB/ITO[e]	-0.1	0.01-5	0.0016	3.2	8
PB/N,P,S@CS/GCE[f]	-0.174	0.0004-2	n.a.	0.2	9
PB-CNTs-paper	0.0	0.1-0.9	0.065	0.48	This work
PB-GR-paper	0.0	0.1-0.9	0.053	0.55	
PB-CF-paper	0.0	0.1-0.9	0.053	0.16	

PB/PET[a] : prussian blue modified polyethylene terephthalate

PB/3DGrE[b] : prussian blue modified 3D printed graphene electrodes

PBNPs/SPEs[c] : prussian blue nanoparticles modified screen printed electrodes

PB/SPCE[d] : prussian blue ink modified screen printed carbon electrode

PB/ITO[e] : prussian Blue modified ITO nanoparticles

PB/N,P,S@CS/GCE[f] : prussian blue electrodeposition on nitrogen, phosphorus and sulfur co-doped porous carbons-chitosan on the glass carbon electrode

Table S3. Comparison of different flexible PEDOT modified electrodes used in the capacitance studied.

Modified electrodes	Capacitance (mF cm^{-2})	Ref.
PEDOT/rGO/PE[a]	18	10
PEDOT:PSS/MEC[b]	6.4	11
PEDOT:PSS/PVA/PMAA[c]	7.38	12
H-TiO_2NTs/pEDOT:Fehcf[d]	26.1	13
PEDOR-CNTs-paper	12.34	This work
PEDOT-GR-paper	12.49	
PEDOT-CF-paper	6.89	

PEDOT/rGO/PE[a] : poly(3,4-ethylenedioxythiophene) polymerized with reduced graphene oxide on poly(ethylene terephthalate)

PEDOT:PSS/MEC[b] : poly(3,4-ethylenedioxythiophene):polystyrene sulfonate printed on micro-scale electrochemical capacitors

PEDOT:PSS/PVA/PMAA[c] : poly(3,4-ethylenedioxythiophene)-poly(styrenesulfonate)/poly(vinyl alcohol)/poly(methacrylic acid)

H-TiO_2NTs/pEDOT:Fehcf[d] : poly(3,4-ethylenedioxythiophene) modified by Prussian Blue analogues deposited onto titania nanotubes and poly(3,4-ethylenedioxythiophene) and iron hexacyanoferrate centres

References

(1) Jiao, L.; Chua, Z. Y.; Moon, S. K.; Song, J.; Bi, G.; Zheng, H.; Lee, B.; Koo, J. Laser-Induced Graphene on Additive Manufacturing Parts. *Nanomaterials*. **2019**, *9*(1), 90.

(2) Banciu, C.; Lungulescu, E. M.; Bara, A.; Leonat, L.; Teisanu, A. 3D graphene network investigation by Raman spectroscopy. *Optoelectron. Adv. Mater. Rapid Commun*. **2017**, *11*, 368-372.

(3) Bourquard, F.; Bleu, Y.; Loir, A. S.; Munoz, B. C.; Avila, J.; Asensio, A.; Raimondi, G.; Shokouhi, M.; Rassas, I.; Farre, C.; Chaix, C.; Barnier, V.; Renault, N. J.; Garrelie, F.; Donnet, C. Electroanalytical Performance of Nitrogen-Doped Graphene Films Processed in One Step by Pulsed Laser Deposition Directly Coupled with Thermal Annealing. *Materials*. **2019**, *12*(4), 666.

(4) Rojas, D.; Rodriguez, J. F.; Pelle, F. D.; Carlo, M. D.; Compagnone, D.; Escarpa, A. Oxidative stress on-chip: Prussian blue-based electrode array for in situ detection of H2O2 from cell populations. *Biosens. Bioelectron.* **2020**, *170*, 112669.

(5) Katic, V.; Santos, P. L.; Santos, M. F.; Pires, B. M.; Loureiro, H. C.; Lima, A. P.; Queiroz, J. C. M.; Landers, R.; Munoz, R. A. A.; Bonacin, J. A. 3D Printed Graphene Electrodes Modified with Prussian Blue: Emerging Electrochemical Sensing Platform for Peroxide Detection. *ACS Appl. Mater. Interfaces***. 2019**, *11*(38), 35068-35078.

(6) Cinti, S.; Arduini, F.; Moscone, D.; Palleschi, G.; Killard, A. J. Development of a Hydrogen Peroxide Sensor Based on Screen-Printed Electrodes Modified with Inkjet-Printed Prussian Blue Nanoparticles. *Sensors*. **2014**, *14*(8), 14222-14234.

(7) Hu, J.Y.; Lin, Y. P.; Liao, Y. C. Inkjet Printed Prussian Blue Films for Hydrogen Peroxide Detection. *Anal. Sci.* **2012**, *28*(2), 135-135.

(8) Ruiz-Vega, G.; Kitsara, M.; Pellitero, A.; Baldrish, E.; Campo, F. J. Electrochemical Lateral Flow Devices: Towards Rapid Immunomagnetic Assays. *ChemElectroChem*. **2017**, *4*(4), 880-889.

(9) Zhai, X.; Li, Y.; Li, J.; Yue, C.; Lei, X. Electrochemical sensor for detection of hydrogen peroxide modified with prussian blue electrodeposition on nitrogen, phosphorus and sulfur co-doped porous carbons-chitosan. *Mater. Sci. Eng. C.* **2017**, *77*, 1242-1246.

(10) Lehtimäki, S.; Suominen, M.; Damlin, P.; Tuukkanen, S.; Kvarnstrom, C.; Lupo, D. Preparation of Supercapacitors on Flexible Substrates with Electrodeposited PEDOT/Graphene Composites. *ACS Appl. Mater. Interface.* **2015**, *7*(40), 22137-22147.

(11) Fan, L.; Zhang, N.; Sun, K. Flexible patterned micro-electrochemical capacitors based on PEDOT. *Chem. Comm*. **2014**, *50*(51), 6789-6792.

(12) Shih, C.C.; Lin, Y. C.; Gao, M.; Wu, M.; Hsieh, H. C.; Wu, N. L.; Chen, W. C. A rapid and green method for the fabrication of conductive hydrogels and their applications in stretchable supercapacitors. *J. Power Sources*. **2019**, *426*, 205-215.

(13) Szkoda, M.; Trzcinski, K.; Rysz, J.; Gazda, M.; Siuzdak, K.; Oleksiak, A. L. Electrodes consisting of PEDOT modified by Prussian Blue analogues deposited onto titania nanotubes – their highly improved capacitance. *Solid State Ion*. **2017**, *302*, 197-201.
